# Dynamics of Neutralizing Antibody Titers in the Months After Severe Acute Respiratory Syndrome Coronavirus 2 Infection

**DOI:** 10.1093/infdis/jiaa618

**Published:** 2020-09-30

**Authors:** Katharine H D Crawford, Adam S Dingens, Rachel Eguia, Caitlin R Wolf, Naomi Wilcox, Jennifer K Logue, Kiel Shuey, Amanda M Casto, Brooke Fiala, Samuel Wrenn, Deleah Pettie, Neil P King, Alexander L Greninger, Helen Y Chu, Jesse D Bloom

**Affiliations:** 1 Division of Basic Sciences and Computational Biology Program, Fred Hutchinson Cancer Research Center, Seattle, Washington, USA; 2 Department of Genome Sciences, University of Washington, Seattle, Washington, USA; 3 Medical Scientist Training Program, University of Washington, Seattle, Washington, USA; 4 Division of Allergy and Infectious Diseases, University of Washington, Seattle, Washington, USA; 5 Department of Biochemistry, University of Washington, Seattle, Washington, USA; 6 Institute for Protein Design, University of Washington, Seattle, Washington, USA; 7 Department of Laboratory Medicine and Pathology, University of Washington School of Medicine, Seattle, Washington, USA; 8 Vaccine and Infectious Disease Division, Fred Hutchinson Cancer Research Center, Seattle, Washington, USA; 9 Howard Hughes Medical Institute, Seattle, Washington, USA

**Keywords:** SARS-CoV-2, spike, RBD, COVID-19, neutralizing antibodies, antibody dynamics

## Abstract

Most individuals infected with severe acute respiratory syndrome coronavirus 2 (SARS-CoV-2) develop neutralizing antibodies that target the viral spike protein. In this study, we quantified how levels of these antibodies change in the months after SARS-CoV-2 infection by examining longitudinal samples collected approximately 30–152 days after symptom onset from a prospective cohort of 32 recovered individuals with asymptomatic, mild, or moderate-severe disease. Neutralizing antibody titers declined an average of about 4-fold from 1 to 4 months after symptom onset. This decline in neutralizing antibody titers was accompanied by a decline in total antibodies capable of binding the viral spike protein or its receptor-binding domain. Importantly, our data are consistent with the expected early immune response to viral infection, where an initial peak in antibody levels is followed by a decline to a lower plateau. Additional studies of long-lived B cells and antibody titers over longer time frames are necessary to determine the durability of immunity to SARS-CoV-2.

## Teaser

After severe acute respiratory syndrome coronavirus 2 infection, neutralizing antibody titers peak about 1 month after symptom onset, then decline about 4-fold over the next 2–3 months, with dynamics typical of immune responses to other respiratory viruses.

Within a few weeks of being infected with severe acute respiratory syndrome coronavirus 2 (SARS-CoV-2), individuals develop antibodies that bind to viral proteins [1–8]. A few weeks after symptom onset, serum from most infected individuals can bind to the viral spike protein and neutralize infection in vitro [5, 7, 9]. The reciprocal dilution of serum required to inhibit viral infection by 50% (neutralizing antibody titer at 50% inhibition [NT_50_]) is typically in the range of 100–200 at 3–4 weeks after symptom onset [10], although neutralizing titers range from undetectable to >10 000 [2, 5, 9].

There are currently limited data on the dynamics of neutralizing antibodies in the months after recovery from SARS-CoV-2. For most acute viral infections, neutralizing antibodies rapidly rise after infection owing to a burst of short-lived antibody-secreting cells and then decline from this peak before reaching a stable plateau that can be maintained for years to decades by long-lived plasma and memory B cells [11, 12]. These dynamics have been observed for many viruses, including influenza [13], respiratory syncytial virus [14], Middle East respiratory syndrome coronavirus [15], SARS coronavirus 1 [16, 17], and the seasonal human coronavirus 229E [18].

Several recent studies have tracked antibody levels in individuals who have recovered from infection with SARS-CoV-2 for the first few months after symptom onset [5, 7, 8, 19–22]. Most of these studies have reported that over the first 3 months, antibodies targeting the spike protein decline several-fold from a peak reached a few weeks after symptom onset [5, 7, 19], suggesting that the early dynamics of the antibody response to SARS-CoV-2 are similar to those for other acute viral infections.

Here we build on these studies by measuring both the neutralizing and binding antibody levels in serial plasma samples from 32 SARS-CoV-2–infected individuals across a range of disease severity with follow-up as long as 152 days after symptom onset. On average, neutralizing titers decreased about 4-fold from approximately 30 to >90 days after symptom onset. This decline in neutralizing titers was paralleled by a decrease in levels of antibodies that bind the spike protein and its receptor-binding domain (RBD). Nonetheless, most recovered individuals still had substantial neutralizing titers at 3–4 months after symptom onset.

## METHODS

### Study Population

Plasma samples were collected as part of a prospective longitudinal cohort study of individuals with SARS-CoV-2 infection. Individuals aged ≥18 years with laboratory-confirmed SARS-CoV-2 infection were eligible for inclusion. Individuals who were human immunodeficiency virus positive were excluded from this study owing to concerns that antiretroviral treatment may affect our pseudotyped lentivirus neutralization assay. Individuals were recruited from 3 groups: inpatients, outpatients, and asymptomatic individuals. Inpatients were hospitalized at Harborview Medical Center, University of Washington Medical Center, or Northwest Hospital in Seattle, Washington, and were enrolled while hospitalized. Outpatients were identified through a laboratory alert system, email and flyer advertising, and through identification of positive coronavirus disease 2019 cases reported by the Seattle Flu Study [23]. Asymptomatic individuals in this study were recruited through outpatient testing and identified when they answered “None” on their symptom questionnaire. They were confirmed to be symptom free for the first 30 days after diagnosis.

We initially enrolled 34 individuals after reverse-transcription quantitative polymerase chain reaction (RT-qPCR) confirmation of SARS-CoV-2 infection. Two individuals (participant identifiers [PIDs] 19C and 196C) were seronegative at all time points in the neutralization assay and all RBD and spike protein enzyme-linked immunosorbent assays (ELISAs) (Supplementary Figure 1). We then tested these individuals using the Abbott Architect antinucleoprotein assay, with which they were also seronegative, with index values of 0.01 (for both samples from PID 196C) or 0.02 (for both samples from PID 19C), far below the threshold for seropositivity of 1.40 [24]. Because these individuals had only a single positive RT-qPCR result (and PID 196C tested negative in 6 subsequent RT-qPCR tests conducted within 15 days of the initial test) and because the Abbott Architect assay has been validated to have very high (95.1%–100%) sensitivity by day 17 after symptom onset [24], we assessed that these 2 individuals were likely not truly infected but rather false-positives in a single RT-qPCR viral test. Therefore, they were excluded from all further analyses, resulting in a final cohort of 32 individuals.

Participants or their legally authorized representatives completed electronic informed consent. Sociodemographic and clinical data were collected from electronic chart review and from participants via a data collection questionnaire (Project REDCap [25]) at the time of enrollment. The questionnaire collected data on the nature and duration of symptoms, medical comorbid conditions, and care-seeking behavior (Supplementary Table 1). Based on these data, individuals were classified by disease severity as asymptomatic, symptomatic nonhospitalized, or symptomatic hospitalized.

Individuals who were recruited as inpatients were enrolled during their hospital admission and had samples collected during their hospitalization. After hospital discharge, these participants subsequently returned to an outpatient clinical research site approximately 30 days after symptom onset for follow-up. In-person follow-up occurred only if participants were asymptomatic, per Centers for Disease Control and Prevention guidelines. Outpatients and asymptomatic individuals completed their enrollment, data collection questionnaire, and first blood sample collection at an outpatient visit approximately 30 days after symptom onset (or positive test for asymptomatic individuals). All participants subsequently were asked to return on day 60 and then on day 90 or 120 for follow-up.

The majority of samples collected from participants were from outpatient visits after recovery. However, the first sample from PID 13, the first 3 from PID 23, and the first 6 from PID 25 were collected during their hospitalizations.

**Figure 1.**
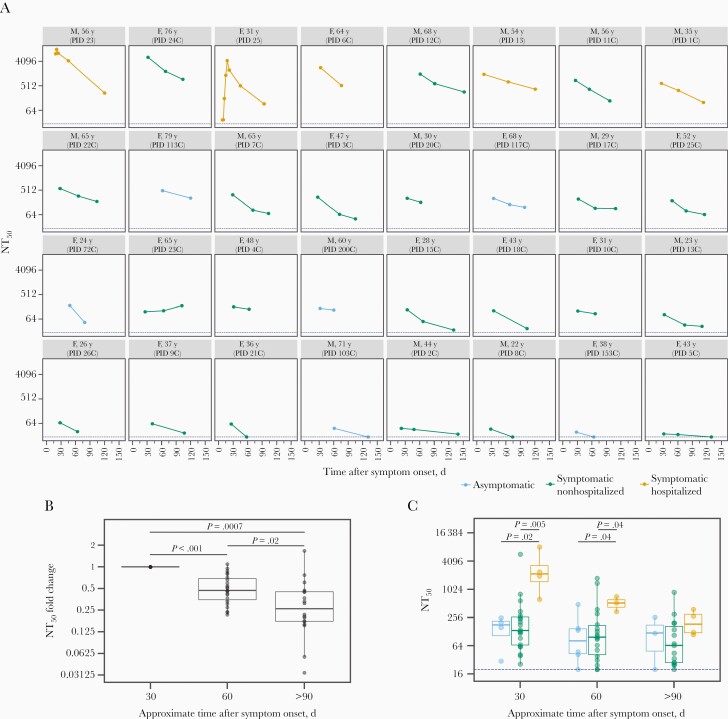
Change in neutralizing antibody titer over time. *A,* Neutralizing antibody titer at 50% inhibition (NT_50_) for each individual in the study, with facets colored according to disease severity (see key below plot). Facet titles indicate sex (F, female; M, male), age, and participant identifier (PID). Dashed blue line indicates the limit of detection for our assay (NT_50_ = 20). *B,* Fold change in NT_50_ compared with 30-day time point, including only individuals with a neutralizing sample at day 30. *P* values were calculated using the Wilcoxon signed rank test. *C,* Distribution of NT_50_ values at the 3 time points, with box plots colored by disease severity and the blue dashed line indicating the limit of detection as in panel A. *P* values are indicated when there is a significant difference (P ≤* *0.05) between NT_50_ values for different disease severity categories at a time point and were calculated using the Wilcoxon rank sum test.

**Figure 2.**
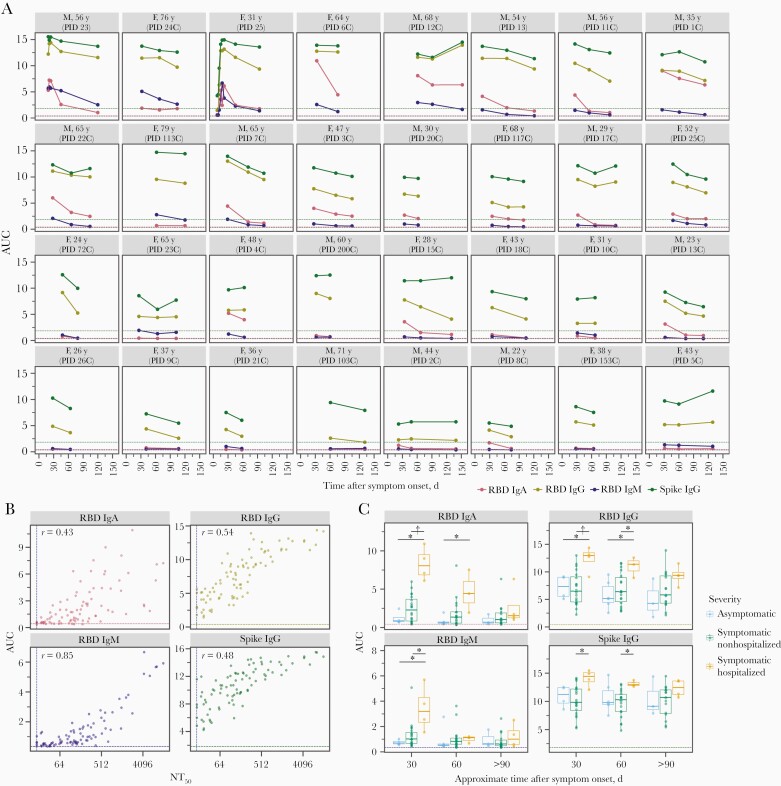
Immunoglobulin (Ig) A, IgM, and IgG antibody binding titers over time. *A,* Longitudinal binding antibody titers for each individual as quantified by area under the curve (AUC) of enzyme-linked immunosorbent assays (ELISAs). Facets are arranged by maximal neutralizing antibody titer at 50% inhibition (NT_50_) from top left to bottom right, as in Figure 1A. Dashed lines indicate the AUC value for the negative control sample (2017–2018 serum sample pool) for each assay, colored by assay. Abbreviations: F, female; M, male; PID, participant identifier; RBD, receptor-binding domain. *B,* Correlation plots between AUC for each ELISA and neutralization titer (NT_50_) for all samples. Vertical dashed line indicates the limit of detection for the neutralization assay; horizontal dashed lines, the AUC values for the negative control sample (2017–2018 serum sample pool) for each assay, colored based on the assay as in *A. C,* For each antibody type measured, individuals who were symptomatic and required hospitalization as part of their care had significantly higher antibody levels during the first 1–2 months after symptom onset. *P ≤ .05; †*P* ≤ .01. *P* values were calculated using the Wilcoxon rank sum test. As in *A* and *B,* the dashed line in each facet indicates the AUC value for the negative control sample for each assay, colored by assay.

For some of the analyses shown in Figures 1 and 2, samples from individuals were divided into 3 time points: approximately 30 days after symptom onset (or after positive test for asymptomatic individuals; range, 22–48 days), approximately 60 days after symptom onset or positive test (range, 55–79 days), and >90 days after symptom onset or positive test (range, 94–152 days). Three individuals (PIDs 2C, 23, and 25) had multiple samples in the first time range. For aggregated analyses that required samples be divided into these 3 groups, we included only the sample closest to 30 days after symptom onset for those individuals. No individuals had multiple samples collected approximately 60 days after symptom onset. One individual (PID 12C) had 2 samples collected >90 days after symptom onset. For this individual, we included only the latest sample collected in aggregated analyses that required classification into groups. The numbers of samples at each time point overall and for each disease severity classification are shown in Table 1. For analyses of fold change, we required individuals to have a sample collected at the 30-day time point. The numbers of individuals included in the fold-change analyses are also indicated in Table 1. This study was approved by the University of Washington Human Subjects Institutional Review Board.

**Table 1.** Demographic Characteristics of Study Participants by Disease Severity Categories

Characteristic	Participants			
	Asymptomatic (n = 6)	Symptomatic Nonhospitalized (n = 21)	Symptomatic Hospitalized (n = 5)	Overall (n = 32)
Age, median (range), y	64 (24–79)	43 (22–76)	54 (31–64)	45.5 (22–79)
Sex, no. (%)				
Male	2 (33.3)	9 (42.9)	3 (60.0)	14 (43.8)
Female	4 (66.7)	12 (57.1)	2 (40.0)	18 (56.3)
Samples per participant, median (range)	2 (2–3)	3 (2–3)	3 (2–8)	3 (2–8)
Samples at each time point (samples included in fold-change analyses), no.				
~30 d	4 (4)	20 (20)	4 (4)	28 (28)
~60 d	6 (4)	18 (17)	3 (2)	27 (23)
>90 d	3 (1)	15 (14)	4 (3)	22 (18)
Duration of follow-up, median (range), d^a^	89 (60–131)	104 (58–152)	113 (76–121)	104 (58–152)

^a^Days between symptom onset date and collection date of last sample. For asymptomatic individuals, test date—calculated as Wednesday of the week of reverse-transcription quantitative polymerase chain reaction test—was used in place of symptom onset date.

### Laboratory Methods

Whole-blood samples were collected in acid citrate dextrose tubes and then spun down, aliquoted, and frozen at −20ºC within 6 hours of collection. Before use in this study, plasma samples were heat inactivated at 56ºC for 60 minutes and stored at 4ºC. Some samples from the early time points were stored at −80ºC after heat inactivation and underwent no more than 2 freeze-thaw cycles. Plasma samples were spun at 2000*g* for 15 minutes at 4ºC immediately before use to pellet platelets.

### Protein Expression and Purification

SARS-CoV-2 RBD and spike (S-2P trimer [26]) proteins were produced in mammalian cells, as described elsewhere [26–28]. Proteins were purified from clarified supernatants as described elsewhere [28]. Sodium dodecyl sulfate–polyacrylamide gel electrophoresis was used to assess purity before flash freezing and storage at −80°C.

### Abbott Architect

Testing of serum samples with the Abbott Architect SARS-CoV-2 immunoglobulin (Ig) G assay was performed according to the manufacturer’s instructions for use and as described elsewhere [24]. Index values associated with the Abbott test are chemiluminescent signal values relative to a calibrator control and are broadly similar to optical density values for an ELISA. An index value ≥1.40 is qualitatively reported as positive.

### ELISAs

The IgG ELISAs for spike protein and RBD were conducted as described elsewhere [27], and were based on a published protocol that recently received emergency use authorization from New York State and the Food and Drug Administration (FDA) [29, 30]. Plasma samples were diluted with 5 serial 3-fold dilutions in phosphate-buffered saline with 0.1% Tween nonfat dry milk, starting at a 1:25 dilution. Each plate contained a negative control dilution series of pooled human serum samples collected in 2017–2018 (Gemini Biosciences; nos. 100–110, lot H86W03J; pooled from 75 donors) and a CR3022 monoclonal antibody positive control dilution series starting at 1 μg/mL.

IgA and IgM RBD ELISAs were performed as described elsewhere for IgG ELISAs [27], with the following changes. The IgA secondary antibody was Peroxidase AffiniPure Goat Anti-Human Serum IgA, α-chain specific (The Jackson Laboratory; no. 109-035-011), and the IgM secondary antibody was goat Anti-Human IgM (μ-chain specific)−Peroxidase antibody (Sigma Aldrich; A6907); both were diluted 1:3000 in phosphate-buffered saline–Tween containing 1% milk. For these ELISAs, plasma samples were analyzed at 6 serial 4-fold dilutions, starting at a 1:25 dilution, again with each plate containing a negative control dilution series (pooled human serum samples obtained in 2017–2018). The area under the curve (AUC) was calculated as the area under the titration curve after putting the serial dilutions on a log-scale.

### Neutralization Assays

Neutralization assays were conducted using pseudotyped lentiviral particles, as described elsewhere [31], with a few modifications. First, we used a spike protein with a cytoplasmic tail truncation that removes the last 21 amino acids (spike ∆21). The map for this plasmid, HDM-SARS2-Spike-delta21, is in Supplementary File 1, and the plasmid is available from Addgene (plasmid no. 155130). We used a spike protein with a C-terminal deletion because, since publication of our original protocol [31], other groups have reported that deleting the spike protein’s cytoplasmic tail improves titers of spike-pseudotyped viruses [32–35]. Indeed, we found that the C-terminal deletion increased the titers of our pseudotyped lentiviral particles without affecting neutralization sensitivity (Supplementary Figure 2).

For our neutralization assays, we seeded black-walled, clear bottom, poly-L-lysine coated 96-well plates (Greiner; no. 655936) with 1.25 × 10^4^ 293T-ACE2 (NR-52511) cells per well in 50 μL of D10 medium (Dulbecco modified Eagle medium with 10% heat-inactivated fetal bovine serum, 2 mmol/L L-glutamine, 100 U/mL penicillin, and 100 μg/mL streptomycin) at 37ºC with 5% carbon dioxide. About 12 hours later, we diluted the plasma samples in D10, starting with a 1:20 dilution followed by 6 or 11 serial 3-fold dilutions (11 dilutions were used for samples from individuals in whom we had previously measured high neutralizing antibody responses; PIDs 13, 23, and 25). We then diluted the spike-∆21 pseudotyped lentiviral particles 1:6 (1 mL of virus plus 5 mL of D10 per plate) and added a volume of virus equal to the volume of plasma dilution to each well of the plasma dilution plates. We incubated the virus and plasma for 1 hour at 37ºC and then added 100 μL of the virus-plus-plasma dilutions to the cells.

At 50–52 hours after infection, luciferase activity was measured using the Bright-Glo Luciferase Assay System (Promega; E2610) as described elsewhere [31], except that luciferase activity was measured directly in the assay plates. Two “no-plasma” wells were included in each row of the neutralization plate, and the fraction infectivity was calculated by dividing the luciferase readings from the wells with plasma by the average of the no-plasma wells in the same row. After calculating fraction infectivities, we used the neutcurve Python package (https://jbloomlab.github.io/neutcurve/) to calculate the plasma dilution that inhibited infection by 50% (IC_50_), fitting a Hill curve with the bottom fixed at 0 and the top at 1. The NT_50_ for each plasma sample was calculated as the reciprocal of the IC_50_. Individuals whose plasma was not sufficiently neutralizing to interpolate an IC_50_ using the Hill curve fit were assigned an NT_50_ of 20 (the limit of our dilution series) for plotting (Figures 1A, 1C, and 2B) and for fold-change analyses (Figure 1B).

All samples were assayed at least in duplicate. We analyzed all samples from the same individual in the same batch of neutralization assays and on the same plate when possible. Each batch of samples included a negative control of pooled serum samples collected from 2017–2018 (Gemini Biosciences; nos. 100–110, lot H86W03J; pooled from 75 donors), and 1 plasma sample known to be neutralizing (from PID 4C at the 30-day time point). These samples were used to confirm consistency between batches.

Results from SARS-CoV-2 spike-pseudotyped lentivirus neutralization assays have been shown to correlate well with full virus SARS-CoV-2 neutralization assays [36, 37]. Nonetheless, in an effort to help standardize comparisons between neutralization assays, we also performed our assay with a standard serum sample from the National Institute for Biological Standards and Control (NIBSC) (Research Reagent for Anti-SARS-CoV-2 Ab; NIBSC code 20/130). This sample had an NT_50_ of approximately 3050 (Supplementary Figure 3).

### Data Availability

Raw data for each sample, including IC50, NT_50_, AUC, and relevant demographic data (age, sex, disease severity, days after symptom onset) are available in Supplementary File 2. Clinical data were analyzed using R software, version 3.6.0 (2019).

## RESULTS

### Longitudinal Plasma Samples From a Cohort of Sars-CoV-2 Infected Individuals

We enrolled 32 individuals after RT-qPCR–confirmed SARS-CoV-2 infection, of whom 5 were symptomatic hospitalized, 21 were symptomatic nonhospitalized, and 6 were asymptomatic (Table 1 and Supplementary Table 1). This cohort included slightly more female than male participants (56.3% female overall), with ages ranging from 22 to 79 years. The age and sex distributions, overall and based on disease severity, are provided in Table 1. Four individuals had comorbid conditions. Information on participant race or ethnicity, symptoms, comorbid conditions, and level of medical care required is provided in Supplementary Table 1.

At least 2 samples were collected from all individuals in this study (median, 3 samples) with the last sample collected 58–152 days after symptom onset (median, 104 days). The majority of individuals (22 of 32) had their last sample collected >90 days after symptom onset.

### Dynamics of Neutralizing Antibody Titers Over Time

We used spike-pseudotyped lentiviral particles [31] to measure neutralizing antibody titers in the longitudinal plasma samples from all 32 infected individuals (Figure 1A). All individuals had detectable neutralizing antibody titers (NT_50_ >20) in their first convalescent plasma sample, which was generally collected roughly 1 month after symptom onset. These data are consistent with prior studies showing that most SARS-CoV-2–infected individuals develop neutralizing antibodies [5, 7, 9]. Qualitative inspection of Figure 1A shows that these titers modestly decreased for most individuals over the next few months, although the dynamics were highly heterogeneous across individuals.

To quantify the dynamics of neutralizing antibody titers over time, we calculated the fold change at approximately 60 and >90 days after symptom onset, relative to the approximately 30-day time point, excluding any individuals who lacked a 30-day sample. Taken across all individuals, neutralizing titers significantly declined from 30 to 60 days, and again from 60 to 90 days (see legend to Figure 1B for details). At >90 days, the median neutralizing titer was reduced 3.8-fold relative to the 30-day value (Figure 1B). However, most individuals (27 of 32) still had detectable neutralizing titers at the last time point.

We compared the dynamics of neutralizing antibody titers between individuals with different disease severities (Figure 1C). Individuals with more severe disease tended to have higher neutralizing antibody titers during early convalescence, consistent with prior studies [5, 38, 39]. Specifically, at both approximately 30 and approximately 60 days after symptom onset, individuals who required hospitalization had significantly higher neutralizing antibody titers than those who did not (Figure 1C). From approximately 30 to >90 days after symptom onset, the NT_50_ for symptomatic hospitalized individuals decreased about 18-fold, significantly more than the approximately 3-fold decrease in the NT_50_ for nonhospitalized individuals (*P = *.03; Wilcoxon rank sum test) (Supplementary Figure 4). By >90 days after symptom onset, neutralization titers did not differ significantly between disease severity groups (Figure 1C). At all time points, asymptomatic individuals had neutralization titers similar to those of symptomatic nonhospitalized individuals.

### Dynamics of Spike Protein–Binding and RBD-Binding Antibodies Over Time

For all plasma samples, we also used ELISAs to measure IgA, IgM, and IgG binding to the RBD of the spike protein, and IgG binding to the full spike protein ectodomain [29]. Figure 2A shows each individual’s IgA, IgM, and IgG binding antibody titers as quantified by the AUC of the ELISA readings (see Methods for detailed description). Like neutralizing antibody titers, these antibody binding titers tended to decrease over time, although there was substantial variation among individuals. All of the ELISA-measured antibody-binding titers are clearly correlated with neutralizing antibody titers (Figure 2B).

Individuals with severe disease had higher antibody binding titers at early time points. Specifically, individuals who were hospitalized as part of their care had higher IgG, IgA, and IgM binding responses than asymptomatic or symptomatic nonhospitalized individuals at approximately 30 days after symptom onset (Figure 2C). By approximately 60 days after symptom onset, anti-RBD IgM levels were no longer significantly different between severity groups, and by >90 days after symptom onset, binding responses did not differ between severity groups for any antibody subtype. This trend is consistent with data in Figure 1C showing that neutralizing antibody responses were higher for individuals with more severe disease early during convalescence but reached similar levels across all disease severity groups by >90 days after symptom onset. Among all patients, regardless of disease severity, IgA and IgM levels decreased more than IgG levels from approximately 30 to >90 days after symptom onset, consistent with findings of other studies [7, 8, 19, 22].

## DISCUSSION

We have measured the dynamics of neutralizing antibody titers over the first 3–4 months after infection with SARS-CoV-2 in a well-characterized prospective longitudinal cohort of individuals across a range of disease severity. The titers of neutralizing antibodies declined modestly, with the titers at 3–4 months after symptom onset generally about 4-fold lower than those at 1 month. This decline in neutralizing antibodies was paralleled by a decline in antibodies binding to the viral spike protein and its RBD. This decline is generally similar in magnitude to that reported in several other recent studies of antibody dynamics in the months immediately after SARS-CoV-2 infection [5, 7, 19].

Individuals with more severe disease tended to have higher peak antibody responses at 1–2 months after symptom onset, consistent with many other studies reporting higher early titers in severely ill SARS-CoV-2–infected individuals [5, 6, 38, 39]. However, by 3–4 months after symptom onset, neutralizing antibody titers among individuals with severe disease were no longer significantly higher than those of individuals with mild symptoms or even asymptomatic infections. Therefore, it seems possible that the large peak in antibody production in severely ill individuals wanes more dramatically than in milder cases, consistent with severe disease often leading to an exaggerated burst of short-lived antibody-secreting cells [40, 41].

Importantly, most individuals in our study still had substantial neutralizing antibody titers at 3–4 months after symptom onset. While some recent studies have interpreted a modest drop in titers in the first few months after infection as alarming, it is entirely consistent with antibody responses to other respiratory viruses. Acute infection is always associated with an initial peak in antibody titers due to a burst of short-lived antibody-secreting cells [42]. For many other infections, titers decline from this initial peak but then reach a stable plateau that is maintained for years or even decades by long-lived plasma cells and memory B cells that can be recalled during subsequent infections [12–16, 18, 43, 44].

The modest declines in antibody titers that we observe over time do have implications for efforts to collect convalescent patient plasma for use in treatment of sick individuals [45]. FDA guidelines suggest minimum cutoffs for the antibody activity in such convalescent plasma (eg, NT_50_ > 160; [39]). Our results suggest that plasma from convalescent donors collected in the first few months after symptom onset will be more likely to meet these cutoffs; others have made a similar observation [46]. In addition, our results indicate that if an individual is donating convalescent plasma over time, each plasma sample should be tested for antibody titers to ensure that they remain above the FDA cutoff.

The limitations of the current study include the small number of samples, particularly in the asymptomatic and symptomatic hospitalized groups, and recruitment of participants from a single study site, which potentially limits the generalizability of these results. Furthermore, since symptom-onset date relies on individual recollections, it is difficult to precisely match the timing of blood draws across all participants. In addition, we had follow-up only to about 4 months after symptom onset, and we only measured plasma antibody responses. Further studies over longer time frames and with direct interrogation of plasma and memory B cells will be necessary to determine longer term durability of immunity to SARS-CoV-2, as well as its relationship to protection against reinfection [47].

Despite these limitations, our study shows that titers of neutralizing and binding antibodies targeting SARS-CoV-2 spike protein remain detectable in most individuals up to >90 days after symptom onset. Although titers decline modestly from approximately 30 to >90 days after symptom onset, we found that the dynamics of the antibody response to SARS-CoV-2 in the first several months after infection are consistent with what would be expected from knowledge of other acute viral infections [13–18].

## Supplementary Data

Supplementary materials are available at The Journal of Infectious Diseases online. Consisting of data provided by the authors to benefit the reader, the posted materials are not copyedited and are the sole responsibility of the authors, so questions or comments should be addressed to the corresponding author.

jiaa618_suppl_Supplementary_Figures

jiaa618_suppl_Supplementary_Table_1

jiaa618_suppl_Supplementary_File_1

jiaa618_suppl_Supplementary_File_2

## Notes

***Acknowledgments.*** We thank Marion Pepper, PhD for helpful input, David Koelle, MD and Anna Wald, MD for sharing reagents, Andrea Loes, PhD and Meei-Li Huang, PhD for experimental assistance, and Ariana Magedson, Dylan McDonald, and Nicholas Franko for assistance with enrollment. We also thank all research participants in the Hospitalized or Ambulatory Adults with Respiratory Viral Infections (HAARVI) study for their generosity in participation.

***Financial support.*** This work was supported by the National Institute of Allergy and Infectious Diseases, National Institutes of Health (grants R01AI141707 to J. D. B. and F30AI149928 to K. H. D. C.), the Bill & Melinda Gates Foundation (H. Y. C.; grant OPP1156262 to N. P. K.), and the Burroughs Wellcome Fund (Investigators in the Pathogenesis of Infectious Disease award to J. D. B.) J.D.B. is an Investigator of the Howard Hughes Medical Institute.

***Conflicts of interest.*** N. P. K. is a cofounder, shareholder, and chair of the scientific advisory board of Icosavax, Inc. A. L. G. has consulted for Abbott Molecular, outside the submitted work. H. Y. C. is a consultant for Merck and Pfizer and receives research funds from Cepheid, Ellume, Genentech, and Sanofi-Pasteur. All other authors report no potential conflicts. All authors have submitted the ICMJE Form for Disclosure of Potential Conflicts of Interest. Conflicts that the editors consider relevant to the content of the manuscript have been disclosed.
