# Supplementary material for: Dynamics of Neutralizing Antibody Titers in the Months After Severe Acute Respiratory Syndrome Coronavirus 2 Infection
Source: J Infect Dis. 2020 Sep 30;223(2):197–205. doi: 10.1093/infdis/jiaa618 (PMC7543487; doi:10.1093/infdis/jiaa618)
Supplement: jiaa618_suppl_Supplementary_Figures [file jiaa618_suppl_supplementary_figures.pdf]

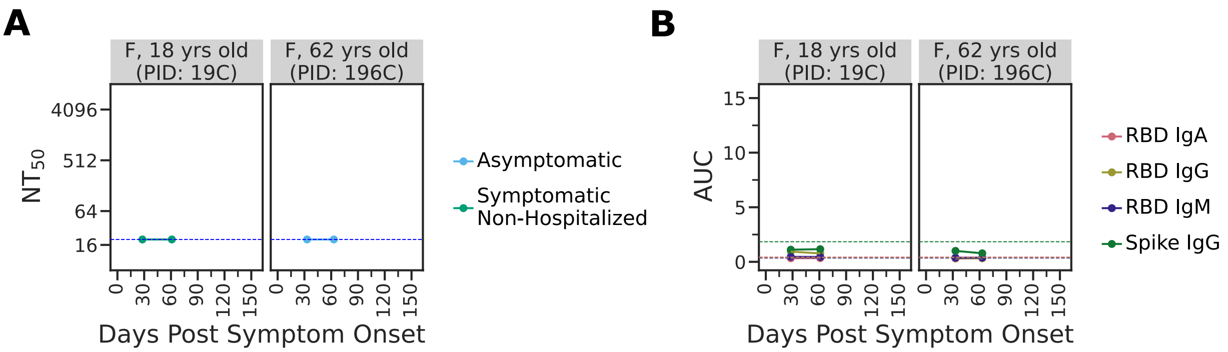

Supplementary Figure 1: Neutralizing and binding antibody levels are not above background for participants 19C and 196C. **A)** Neither PID 19C nor 196C had detectable neutralizing titers at any timepoint. The limit of detection for our neutralization assay (NT_{50} = 20) is shown with a dashed blue line. **B)** Neither PID 19C nor 196C had detectable spike or RBD binding antibodies at any timepoint. The binding levels for the negative control sample (2017-2018 sera pool) are shown as dashed lines colored by assay.

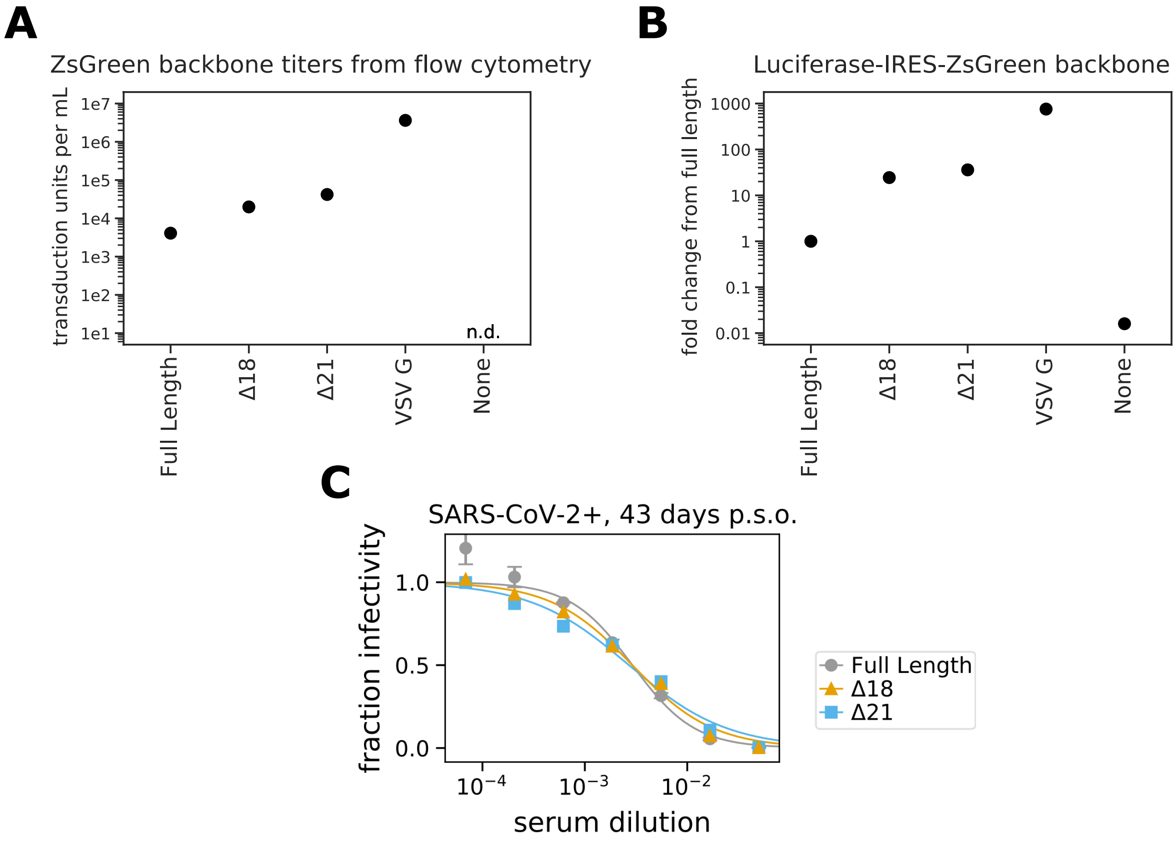

Supplementary Figure 2. Titers of spike-pseudotyped lentiviral particles in 293T-ACE2 cells (BEI Resources, NR-52511) **(A, B)** and neutralization of these viruses with serum from an individual previously infected with SARS-CoV-2 **(C)**. **A)** Titers of pseudotyped lentivirus with a ZsGreen backbone (BEI, NR-52520) pseudotyped with full-length spike (BEI, NR-52514), spike with either the last 18 (Δ18) or 21 (Δ21) amino acids truncated, VSV G, or no viral entry protein. Titers were determined as described in [1]. Positive cells were counted via flow cytometry at 60 h post-infection. The "n.d." indicates that the titer was not detectable. Data shown are from a single representative example. **B)** Titers of Luciferase-IRES-ZsGreen backbone virus pseudotyped with the specified viral entry proteins. Titers were determined by measuring relative luciferase units (RLUs) per mL and then normalizing to the titers of full-length spike pseudotyped lentivirus. RLUs were determined at 52 h post-infection. The RLUs per mL for the spike-pseudotyped viruses are the average of seven wells of a 1:3 dilution of virus in a total volume of 150 µL. For the VSV G-pseudotyped virus, RLUs per mL were averaged from six three-fold dilutions starting at a 1:48 dilution in a total volume of 150 µL. **C)** Neutralization assay with serum collected from an individual previously infected with SARS-CoV-2, 43 days post-symptom onset (p.s.o.). The full-length neutralization curve shows data averaged from duplicate measurements. The Δ18 and Δ21 neutralization curves display data from a single replicate. The IC50s for the full-length, Δ18, and Δ21 viruses are 1:345, 1:345, and 1:370, respectively. These values all fall within the range of IC50 values we have measured previously for this same serum sample with virus pseudotyped with full-length spike (1:320-1:375). We thank Dr. David Koelle and Dr. Anna Wald at the University of Washington for sharing this sample with us.

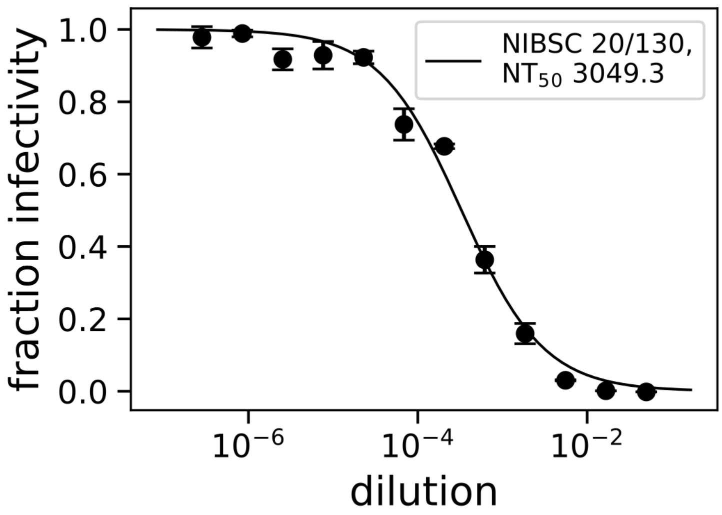

Supplementary Figure 3: Neutralization curve for NIBSC standard reference serum (product number 20/130). The NT_{50} for this sample was calculated to be ~3050.

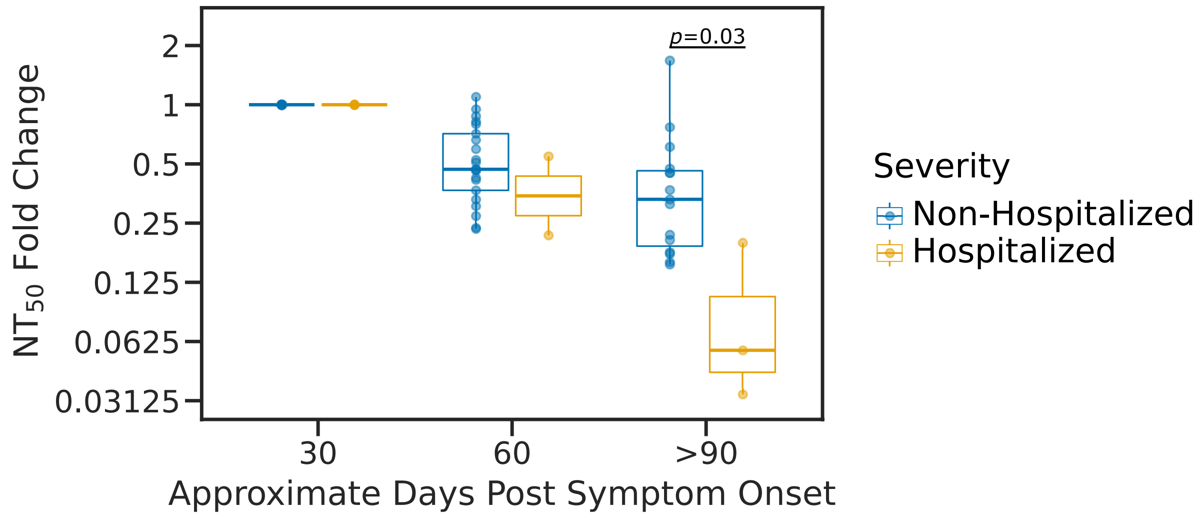

Supplementary Figure 4: Fold change in NT_{50} at each timepoint colored by disease severity. There was only one asymptomatic individual with a 30-day timepoint and 90-day timepoint, so this individual was included in the *Non-Hospitalized* group. As in **Figure 1B**, only individuals with a sample at ~30 days post-symptom onset are included. *P*-value calculated using the Wilcoxon rank-sum test.

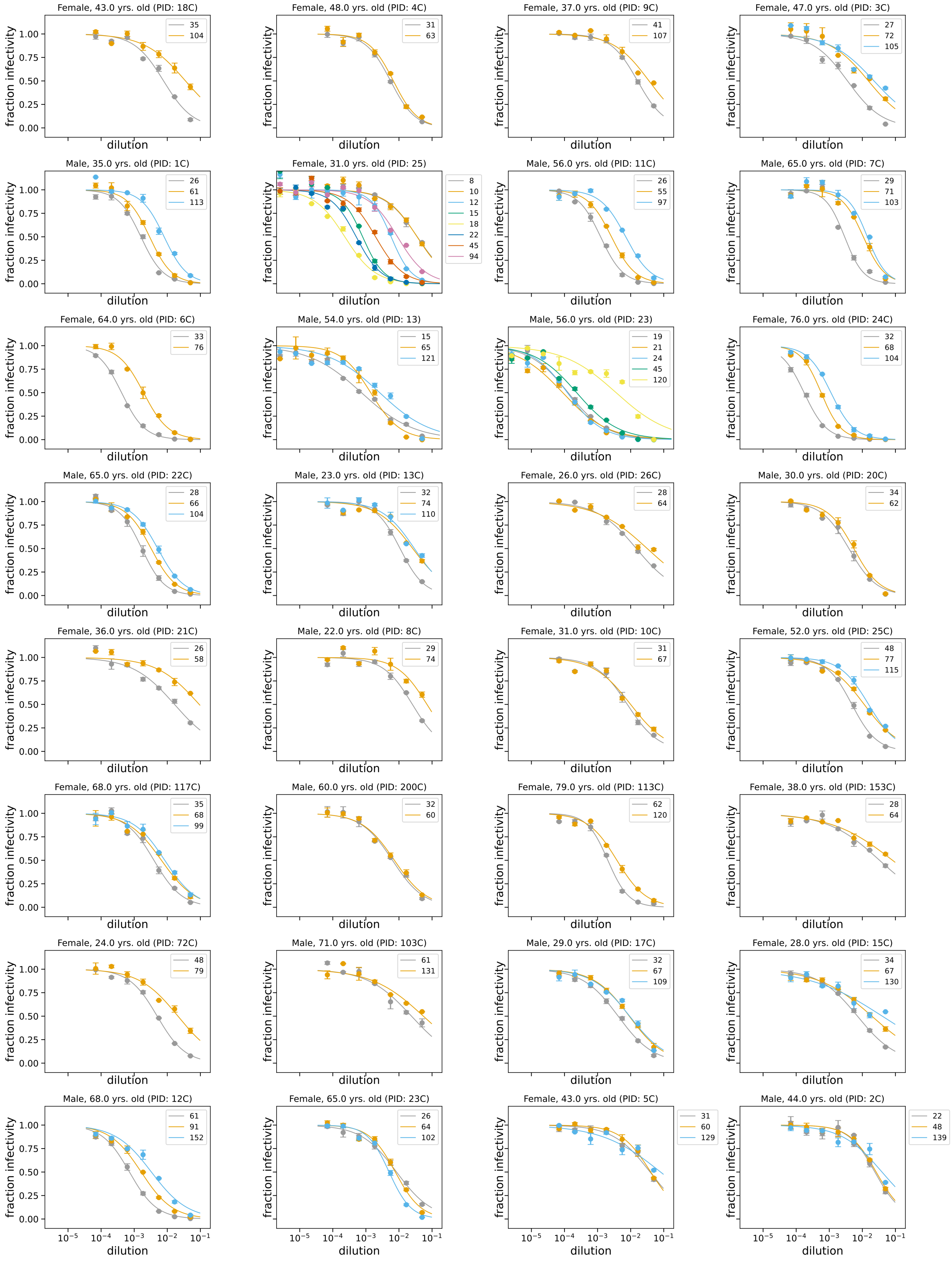

Supplementary Figure 5: Neutralization curves for each participant. For each participant, each sample is a different color with the legend specifying how many days post-symptom onset each sample was collected.

Supplementary File 1: Plasmid map for the spike plasmid (HDM_Spikedelta21) used in the pseudotyped lentivirus neutralization assays.

Supplementary File 2: Raw data for each sample. This csv includes the columns: sample, participant ID, Sex, Age, Severity, Days Post-Symptom Onset, IC50, NT50, RBD IgA, RBD IgG, RBD IgM, and Spike IgG. ELISA results are presented as area under the curve (AUC).

References:

1. Crawford KHD, Eguia R, Dingens AS, et al. Protocol and Reagents for Pseudotyping Lentiviral Particles with SARS-CoV-2 Spike Protein for Neutralization Assays. Viruses [Internet]. **2020**; 12(5). Available from: http://dx.doi.org/10.3390/v12050513
