# Supplementary material for: Dynamics of Neutralizing Antibody Titers in the Months After Severe Acute Respiratory Syndrome Coronavirus 2 Infection
Source: J Infect Dis. 2020 Sep 30;223(2):197–205. doi: 10.1093/infdis/jiaa618 (PMC7543487; doi:10.1093/infdis/jiaa618)
Supplement: jiaa618_suppl_Supplementary_Table_1 [file jiaa618_suppl_supplementary_table_1.pdf]

Supplementary Table 1: Additional demographic and medical data.

	Asymptomatic (N=6)	Symptomatic Non-Hospitalized (N=21)	Symptomatic Hospitalized (N=5)	Overall (N=32)
**Race**				
AIAN	1 (16.7%)	0 (0%)	0 (0%)	1 (3.1%)
White	5 (83.3%)	20 (95.2%)	1 (20.0%)	26 (81.2%)
Asian	0 (0%)	1 (4.8%)	3 (60.0%)	4 (12.5%)
Black	0 (0%)	0 (0%)	0 (0%)	0 (0%)
Multiple Races	0 (0%)	0 (0%)	1 (20.0%)	1 (3.1%)
**Hispanic**	0 (0%)	1 (4.8%)	0 (0%)	1 (3.1%)
**Smoking**				
Nonsmoker	5 (83.3%)	19 (90.5%)	5 (100%)	29 (90.6%)
Tobacco use	1 (16.7%)	2 (9.5%)	0 (0%)	3 (9.4%)
Electronic cigarettes/vapor pen use	0 (0%)	1 (4.8%)	0 (0%)	1 (3.1%)
**Comorbidities**[a]				
No comorbidities	6 (100%)	19 (90.5%)	3 (60.0%)	28 (87.5%)
Asthma	0 (0%)	0 (0%)	1 (20.0%)	1 (2.9%)
Hypertension	0 (0%)	2 (9.5%)	1 (20.0%)	3 (9.4%)
Diabetes	0 (0%)	0 (0%)	1 (20.0%)	1 (3.1%)
COPD/emphysema	0 (0%)	0 (0%)	1 (20.0%)	1 (3.1%)
Cancer	0 (0%)	0 (0%)	1 (20.0%)	1 (3.1%)
Obstructive Sleep Apnoea	0 (0%)	0 (0%)	1 (20.0%)	1 (3.1%)
**Common Symptoms**[a]				
Chills or shivering	0 (0%)	13 (61.9%)	5 (100%)	18 (56.2%)
Cough	0 (0%)	15 (71.4%)	5 (100%)	20 (62.5%)
Diarrhea	0 (0%)	6 (28.6%)	1 (20.0%)	7 (21.9%)
Ear pain or ear discharge	0 (0%)	0 (0%)	0 (0%)	0 (0%)
Fatigue	0 (0%)	16 (76.2%)	1 (20.0%)	17 (53.1%)
Feeling feverish	0 (0%)	13 (61.9%)	5 (100%)	18 (56.2%)
Increased trouble breathing	0 (0%)	4 (19.0%)	5 (100%)	9 (28.1%)

Loss of sense of taste or smell	0 (0%)	7 (33.3%)	0 (0%)	7 (21.9%)
Muscle or body aches	0 (0%)	13 (61.9%)	2 (40.0%)	15 (46.9%)
Nausea or vomiting	0 (0%)	3 (14.3%)	2 (40.0%)	5 (15.6%)
Rash	0 (0%)	1 (4.8%)	0 (0%)	1 (3.1%)
Runny or stuffy nose	0 (0%)	12 (57.1%)	1 (20.0%)	13 (40.6%)
Sore throat or itchy/scratchy throat	0 (0%)	9 (42.9%)	0 (0%)	9 (28.1%)
Sweats	0 (0%)	13 (61.9%)	4 (80.0%)	17 (53.1%)
**Highest Level of Medical Treatment Received**				
Outpatient - Testing Only	5 (83.3%)	13 (61.9%)	0 (0%)	18 (56.2%)
Outpatient - Saw Provider[b]	1 (16.7%)	8 (38.1%)	0 (0%)	9 (28.1%)
Inpatient (General Floor)	0 (0%)	0 (0%)	2 (40.0%)	2 (6.2%)
Inpatient (ICU)	0 (0%)	0 (0%)	3 (60.0%)	3 (9.4%)
**Highest Level of Respiratory Support**				
None	6 (100%)	21 (100%)	0 (0%)	29 (85.3%)
Nasal Cannula	0 (0%)	0 (0%)	1 (20.0%)	1 (2.9%)
High-flow O2 or non-rebreather	0 (0%)	0 (0%)	1 (20.0%)	1 (2.9%)
Non-invasive ventilation (BiPAP)	0 (0%)	0 (0%)	1 (20.0%)	1 (2.9%)
Mechanical Ventilation	0 (0%)	0 (0%)	2 (40.0%)	2 (5.9%)
**Duration of mechanical ventilation (days) (N=2)**				
Median [Min, Max]	NA [NA, NA]	NA [NA, NA]	11.5 [6.00, 17.0]	11.5 [6.00, 17.0]

[a]Categories not mutually exclusive
[b]Includes Primary care physician, Urgent care, Emergency Department
